# The IL-23R and Its Genetic Variants: A Hitherto Unforeseen Bridge Between the Immune System and Cancer Development

**DOI:** 10.3390/cancers17010055

**Published:** 2024-12-27

**Authors:** Salvatore Audia, Carolina Brescia, Vincenzo Dattilo, Naomi Torchia, Francesco Trapasso, Rosario Amato

**Affiliations:** 1 Immuno-Genetics Lab, Department of Health Science, Medical School, University “Magna Graecia” of Catanzaro, 88100 Catanzaro, Italy; salvatore.audia001@studenti.unicz.it (S.A.); brescia@unicz.it (C.B.); naomi.torchia@studenti.unicz.it (N.T.); 2 Department of Experimental and Clinical Medicine, Medical School, University “Magna Graecia” of Catanzaro, 88100 Catanzaro, Italy; dattilo@unicz.it

**Keywords:** IL-23R, immune cells, genetic variants, Th17, tumor microenvironment

## Abstract

IL-23R (interleukin-23 receptor), found on the surface of several immune cells, plays a key role in the immune system. Indeed, this process is not limited to the inflammatory response but also plays a role in the adaptive immune response. The binding between IL-23R and its specific ligand, the interleukin 23, initiates a number of specific signals by modulating both properties and behavior of immune cells. In particular, it is critical for the regulation of T helper 17 cells (Th17). Th17s are a subset of T cells involved in autoimmune and inflammatory diseases, as well as in cancer. The clinical relevance of IL-23R is underscored by its association with an elevated susceptibility or diminished vulnerability to a spectrum of diseases, including psoriasis, ankylosing spondylitis, and inflammatory bowel disease (IBD). Evidence has emerged that suggests it may also serve to predict both tumor progression and therapeutic responsiveness. It is noteworthy that the IL-23/IL-23R pathway is emerging as a promising therapeutic target. A number of biologic drugs, such as monoclonal antibodies, are currently developing with the aim of blocking this interaction, thus reducing inflammation. This represents a significant advancement in the field of medicine, offering new hope for pursuing more effective and personalized treatments. Recent studies have also investigated the role of such a pathway in autoimmune diseases, and its potential impact on infections as well as in carcinogenesis. The aim of this review is to focus on the role of IL-23R in immune genetics and its potential for modulating the natural history of neoplastic disease.

## Simple Summary

The interleukin-23 receptor (IL-23R) is a crucial regulatory element of the immune system with profound implications for human health. Signaling downstream of IL-23R polarizes and directs a broad spectrum of lymphocyte populations, particularly CD4^+^ subsets such as Th17 and Treg, thereby influencing the pro- or anti-inflammatory roles of these cells. The clinical relevance of IL-23R is considerable, with variants in this gene having been associated with an increased risk of developing autoimmune diseases. Furthermore, the emerging role of IL-23R in carcinogenesis is worthy of note. Several variants in the IL-23R gene have been identified as predisposing factors in the development of disparate types of cancer in different populations, while other variants appear to be protective factors. A comprehensive grasp of the mechanisms underlying the IL-23/IL-23R pathway and the capacity to modulate it represent indispensable prerequisites for the formulation of novel therapeutics for the treatment of immune-mediated disorders and cancer. This knowledge promises the emergence of more efficacious and personalized interventions.

## 1. Introduction

**Figure 1:**
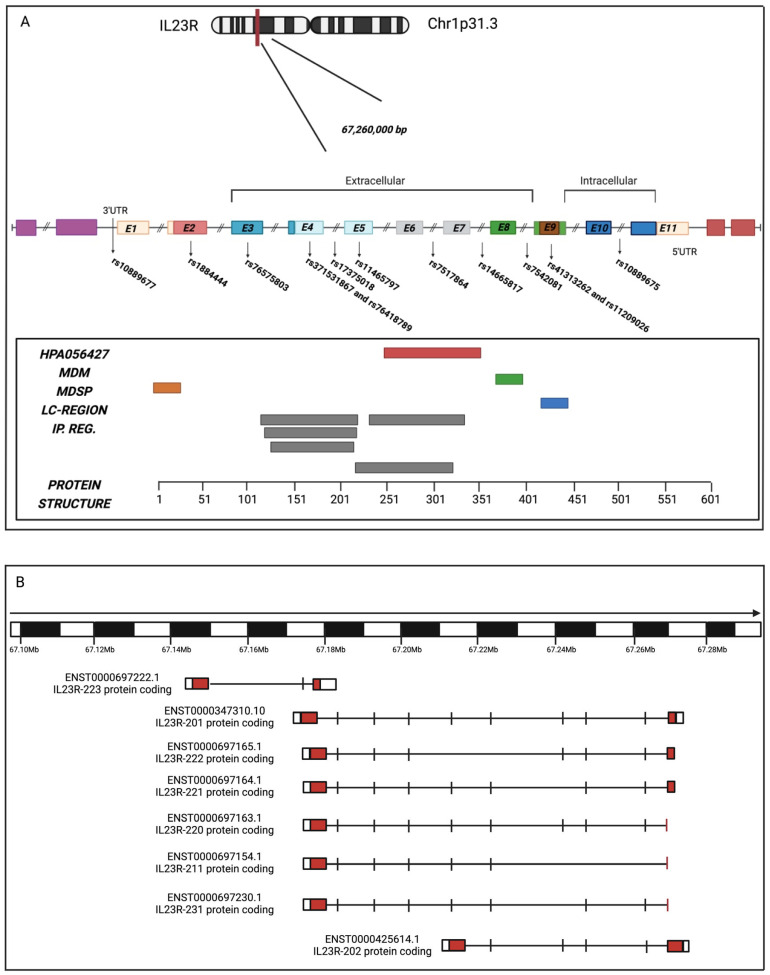
Structure of IL-23R. (**A**). Chromosomal gene mapping, correlated transcription, variants position between exons and introns according to ClinVar, GnomAD and Ensembl. The protein structure is shown below, adapted from the human protein atlas. The main domains are annotated as they are organized in the protein structure: HPA056427 (antigenically recognized domain); MDM and MDSP (trans-membrane portion and localization signal); LC-reg (low complexity region); IP-reg (immunoglobulin-like fold domains and fibronectin III superfamily domains). (**B**). Graphical representation of the major alternative splicing forms of the IL-23R gene by distribution frequency, with gene location and structure. The variants described have been extracted from ensembl.org. Created in BioRender. www.biorender.com/ (accessed on 22 November 2024).

The interleukin-23 receptor is a type I cytokine receptor. Class I and Class II cytokine receptors are single transmembrane domain (TMD)-containing proteins that associate to form homo- or heteromeric receptor complexes that bind cytokine ligands [1]. In humans, the *IL-23R* gene is located on the short arm of chromosome 1 (1p31.3) in close proximity to the gene encoding *interleukin-12 beta receptor subunit 2 (IL-12Rβ2)*. The mRNA encoding IL-23R is 2.8 kilobases in length. The translated protein comprises 629 amino acids and has a molecular weight of 71,722 Da [2] (Figure 1A). IL-23R is integrated into the outermost membrane of various immune system cells, including T cells, natural killer cells, monocytes and dendritic cells. These cells serve to identify foreign substances and thus protect the body against infection and disease [3]. The IL-23R is localized at the cell surface where it exerts its intra- and extracellular effects upon binding to IL-23. IL-23 is a pro-inflammatory cytokine that belongs to the IL-12 family [4]. IL-23 is primarily produced by dendritic cells and activated macrophages. It then exerts its effects on Th17, a distinct subpopulation of gamma/delta T cells, natural killer T cells, and type 3 innate lymphoid cells [5,6]. The pathological consequences of excessive IL-23/IL-23R signaling have been linked to its ability to promote the production of inflammatory mediators, including interleukin-17 (IL-17), interleukin-22 (IL-22), granulocyte-macrophage colony-stimulating factor (GM-CSF) and tumor necrosis factor (TNF-α). The mediators facilitate the recruitment and activation of granulocytes and macrophages, which, in turn, induce damage including chronic inflammation and the development of tumors [6,7,8]. This process also results in the generation of an accompanying tumor microenvironment, which exhibits the following characteristics. First, the IL-23/IL-23R axis has been observed to interfere with the antitumor function of natural killer (NK) cells by blocking the interferon gamma (IFNγ) and perforin-mediated effects. Second, this same axis has been seen to support neoangiogenesis, thereby inhibiting the infiltration of CD8^+^ T cells into the tumor tissue. Third, IL-23 has been shown to activate DNA repair pathways, an activity that occurs via an immune-independent pathway [9]. It has been demonstrated that multiple isoforms of the IL-23R gene may be produced by alternative splicing, resulting in premature termination and frameshifts that can generate receptors lacking essential signaling components, or isolated ectodomains with the capacity to act as decoy receptors [10,11] (Figure 1B). A substantial number of genetic variants have been identified at the IL-23R locus [3] (Figure 1A). The expression of IL-23R can be controlled post-transcriptionally by several miRNAs, thereby introducing another layer of complexity to the regulation of IL-23R.

The microRNA Let-7f has been observed to target the 3′ untranslated region (UTR) of IL-23R and its overexpression has been demonstrated to result in a reduction in IL-23R in human CD4^+^ T cells. The levels of Let-7f can be regulated by estrogen signaling. Indeed, Th17 cells from female asthma patients exhibited lower Let-7f expression and higher IL-23R expression than Th17 cells from male patients. Furthermore, the inhibition of Let-7f led to an additional increase in IL-23R levels [12]. In addition, the overexpression of let-7fg in activated CD4^+^ T cells has been demonstrated to protect against severe disease in a mouse model of multiple sclerosis. This is achieved by reducing the secretion of proinflammatory cytokines and the expression of IL-23R in Th17 cells [13]. Furthermore, additional microRNAs, specifically miR-34a and miR146b-5p, have the potential to target the 3′UTR of IL-23R. In murine models exhibiting deficiency of miR-34a, a notable increase in both IL-23R and IL-17 levels was observed in the colon following infection. It would appear that the anti-inflammatory effect of miR-146b-5p on IL-23R expression in chronic lymphocytic leukemia cells is mediated through the downregulation of the IL-12Rβ1 chain [14,15].

## 2. IL-23/IL-23R Interaction

**Figure 2:**
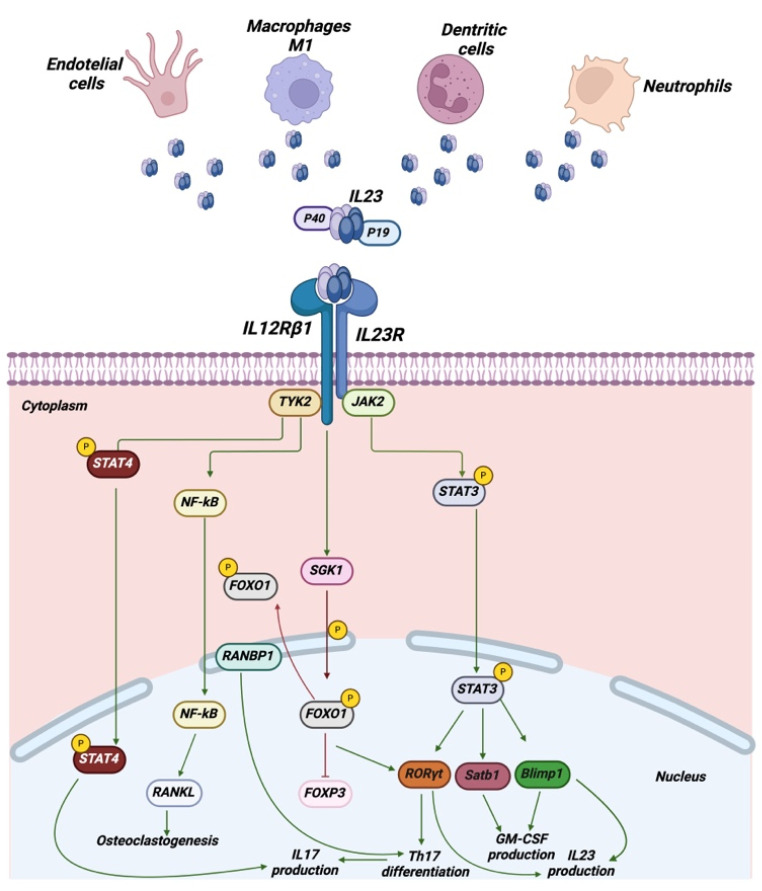
Signaling by IL-23R. Some of the main producers of IL-23 are endothelial cells, macrophages, dendritic cells and neutrophils. Pathways are started when the two subunits of IL-23, p19 and p40, bind to IL-23R and IL-12Rβ1. This activates Tyk2 and Jak2. Tyk2 and Jak2 mainly promote STAT, NF-κB, SGK1, RORγt, Satb1 and Blimp1 and related down-stream effectors. Created in BioRender. www.biorender.com/ (accessed on 20 December 2024).

Upon binding to its receptor, IL-23 initiates a cascade of molecular signals within the cell. These signals promote the inflammatory response and facilitate the coordination of the immune system’s reaction to external pathogens, including bacteria and viruses [6]. IL-23 represents a member of the IL-12 type cytokine family, exhibiting substantial homology with the IL-6 type cytokines. The IL-12 family comprises three heterodimeric cytokines: IL-12, IL-23, and IL-35 [10]. IL-23 is a heterodimeric cytokine, comprising two distinct subunits: p19, encoded by *interleukin 23A (IL-23A)*, and p40, encoded by *interleukin 12B (IL12B)* [16]. The latter subunit is also present in another cytokine, namely interleukin-12 (IL-12) [17]. Furthermore, it should be noted that both IL-23 and IL-12 receptors possess a common chain, namely IL-12Rβ1. This chain forms a complex with the IL-23R chain, which is responsible for conferring responsiveness to IL-23. An alternative configuration is formed when the receptor complex binds with IL-12Rβ2. This complex is known as the IL-12 receptor complex [18,19]. Although they possess shared subunits, IL-23 and IL-12 exhibit disparate and distinctive biological functions [20]. IL-23 binds to a membrane receptor complex consisting of two type I membrane proteins: IL-12Rβ1 and IL-23R. The IL-12Rβ1 subunit comprises two extracellular cytokine receptor domains and three type-III fibronectin domains, which are followed by a single transmembrane domain and a cytosolic domain [21]. The IL-23R subunit contains an N-terminal immunoglobulin-like domain, two cytokine receptor domains, a single transmembrane domain, and a cytosolic domain [2]. It has been demonstrated that the IL-23R chain initially interacts with the IL-23p19 subunit exclusively through its N-terminal domain. This results in a conformational change to the interleukin-23 heterodimer, which permits the p40 subunit to bind to the IL-12Rβ1 with high affinity, thus recruiting the complex [22]. The interaction between p40 and IL-12Rβ1, and p19 and IL-23R is a prerequisite for the formation of a complex competent for signaling, which is composed of the three aforementioned proteins [23] (Figure 2).

## 3. IL-23R Signaling Cascade

It is established that the IL-23 receptor complex associates with multiple members of the Janus kinase (JAK) family, including Jak2 and Tyk2. The IL-12Rβ1 complex recruits TYK2, while the IL-23R complex interacts with JAK2 [24]. The Box1 and Box2 motifs, which are located within the intracellular domain of IL-12Rβ1, have been identified through the use of deletion and site-directed mutagenesis techniques as binding sites for Tyk2. In comparison to other receptors, IL-23R is distinguished by the absence of Box1 and Box2 motifs, which have been identified as atypical JAK2 binding sites within its cytoplasmic tail [25]. The analysis of kinase-deficient cell lines has shown that both Jak2 and Tyk2 are required for the signal transduction of IL-23 [2,25]. Activation of Tyk2 and Jak2 by IL-23R mainly promotes STAT3, but also STAT1, STAT4 and STAT5 phosphorylation [2]. In contrast, IL-12Rβ2-mediated signaling mainly promotes STAT4 phosphorylation. However, it can also induce STAT1 and STAT5 phosphorylation [26,27]. IL-23-mediated activation of STAT3 induces the expression of RORγt (RAR-related orphan receptor gamma), the master regulator of Th17 differentiation. RORγt is critical for Th17 differentiation and IL-17 production [28,29,30]. Additionally, STAT3 has the potential to enhance IL-23R expression, thereby amplifying the feedback that maintains the IL-17/IL-23 pathway [31]. T-bet represents another pivotal gene in the differentiation of Th17 cells. The synthesis of IL-17 and IFNγ is contingent upon this gene, which is stimulated by IL-23 [32]. Specifically, following the binding of IL-23 to its receptor, IL-12Rβ2 undergoes phosphorylation and subsequently functions as a docking site for STAT4. Subsequently, STAT4 binds to the receptor chain and undergoes phosphorylation. STAT4 homodimers are transported into the nucleus, where they bind to STAT-binding sites in the interferon (IFN)-γ promoter, thereby inducing transcription of the IFN-γ gene. IFN-γ can induce the proliferation of CD8^+^ cytotoxic T cells, as well as naïve and memory T cells. Furthermore, it has been observed to have anti-tumor growth and anti-metastasis effects. The pro or anti-tumorigenic effects of the IL-23/IL-23R axis are dependent upon the balance of STAT3 signaling within the tumor and the surrounding tumor cell microenvironment [9]. Additionally, IL-23 has been demonstrated to facilitate the translocation of NF-κB into the nucleus, thereby inducing the expression of the receptor-activating ligand of NF-κB (RANKL), a pivotal osteoclastogenic cytokine implicated in bone destruction and arthritic pathology [33,34,35]. IL-23 has been demonstrated to promote the expression of the transcription factor *Blimp1* (also known as PRDM1) and *Satb1* (Special AT-Rich Sequence-Binding Protein 1) in Th17 cells, which are both necessary for GM-CSF production and IL-23R expression [36,37,38] (Figure 2).

## 4. IL-23R in Immune Cells

The majority of studies that have characterized the IL-23/IL-23R pathway have focused on T lymphocytes, with particular emphasis placed on CD4^+^ T cells. This is due to the fact that the primary function of IL-23 is to regulate the activity of T cells. Dendritic cells, certain leukocyte populations, macrophages and endothelial cells are capable of secreting IL-23. Macrophages and dendritic cells are the primary sources of IL-23, a significant marker of macrophage polarization towards the M1 phenotype. IL-23R is predominantly expressed on the surface of T cells [3,39]. The biological functions of IL-23R in other immune cells remain poorly understood. The current evidence suggests that the majority of immune cells must undergo activation in order to become responsive to IL-23. However, the stimuli and transcriptional pathways that induce IL-23R expression in different cell types are highly variable and dependent on cell type and environmental context, and thus remain poorly characterized [3].

### 4.1. T Cells

IL-23R was first identified in 2002 and has since been the subject of extensive research, particularly with regard to CD4^+^ T cells, especially Th17 cells [2,40]. It was found that while murine double-positive thymocytes express IL-23R at all times, there is no expression of IL-23R in T cells observed in the periphery unless they are first activated [41]. It has been demonstrated that IL-23 can upregulate its own receptor in TCR-stimulated human CD4^+^ T cells, resulting in increased IL-22 and IL-17 secretion [42]. It has previously been demonstrated that the differentiation of naïve CD4^+^ T cells with the cytokine combinations of IL-12 and IL-21 in vitro results in the production of high levels of the IL-23 receptor (IL-23R), which has been shown to contribute to the pathogenesis of Th1-like cells during intestinal inflammation [43]. It has been postulated that interleukin (IL)-6 and transforming growth factor beta (TGF-β) play a substantial role in the induction of interleukin (IL)-23 receptor (IL-23R) in mouse CD4⁺ T cells. Specifically, these two factors have been identified as critical drivers in the development of hyper-inflammatory Th17 cells, which are distinguished by elevated expression of IL-23R [44]. The IL-23/IL-23R pathway has been demonstrated to support the expansion and survival of murine Th17 cells. Furthermore, Th17 clones have been observed to express higher levels of IL-23R transcripts than Th1 cells [45]. Although IL-23R transcripts have been identified in Th1 cell clones, T helper cells undergo overexpression of IL-17A and IL-23R through SGK1 (serum/glucocorticoid regulated kinase 1) activation, resulting in differentiation into Th17 cells. Concurrently, both polarization toward the Th17 phenotype and impairment of Treg suppressor function represent key mechanisms involved in these effects [46,47]. SGK1 is a serine/threonine kinase of the AGC family that plays a role in inflammatory processes, stress responses, and neoplastic development [48,49,50]. SGK1 is currently the leading candidate for the IL-23R-dependent differentiative mechanism in Th17, both in the presence and absence of NaCl stimulation [51,52]. SGK1 plays a pivotal role in the development of the Th17 phenotype [53], which is associated with a heightened risk of developing conditions such as multiple sclerosis, systemic lupus erythematosus, autoimmune colitis and transplant rejection [47]. When the microenvironment stimulates IL-23 production, it binds with IL-23R and activates SGK1, causing phosphorylation of FOXO1 [51,54] and concomitant overexpression of RANBP1, a small GTPase that controls the nucleus-cytoplasmic transition [55,56,57]. RANBP1 is essential for the nucleocytoplasmic translocation and degradation of FOXO1, which acts as a direct antagonist to the RORγt-Th17+ differentiation program [51,58]. This results in a considerable elevation in Th17 differentiation and a comprehensive cessation of FOXP3-dependent Treg differentiation [48,59]. RANBP1 serves as the final indispensable component in the IL-23R-mediated Th17+ differentiation pathway, mediating SGK1-dependent effects [60,61]. It is noteworthy that this signaling pathway appears to be associated with the pathological Th17+ response, which is characterized by the expression of high levels of IL-17 and low levels of IL-10 [51,62]. A subset of regulatory T cells that express the transcription factor RORγt may also be responsive to IL-23 [63]. IL-23R is enriched in intestinal Tregs, which highly express IL-23 compared with Tregs from other compartments. Upon IL-23 stimulation, the frequency of these Tregs is reduced, and their suppressive function is impaired. IL-23R signaling regulates intestinal Tregs by increasing cell turnover, antagonizing suppression and decreasing cholesterol efflux [64]. In an experimental colitis model, IL-23R signaling was observed to decrease the frequency of Tregs in the gut and impair their suppressive functions, potentially due to a reduction in Foxp3 expression [61,65]. Additionally, IL-23 signaling has been demonstrated to facilitate the expansion of regulatory T cells (Tregs) in murine models of dermatological disease. This is characterized by the accumulation of Treg cells that express the transcription factor RORγt and the IL-17, as evidenced by reference [66]. A study utilizing a mouse oral squamous cell carcinoma (OSCC) model revealed that IL-23R^+^ regulatory T cells (Tregs) demonstrated lower expression of the Foxp3 transcription factor and elevated T-bet protein compared to IL-23R- Tregs. Tregs that were IL-23R- produced a substantial amount of IL-10 and TGF-β, while IL-23R^+^ Tregs produced less IL-10 and TGF-β yet a considerably greater amount of IFN-γ. Additionally, IL-23R^+^ Tregs exhibited elevated levels of phosphorylated STAT3 and STAT4 compared to IL-23R- Tregs. IL-23R^+^ Tregs demonstrated a reduced immunosuppressive capacity compared to IL-23R- Tregs [67]. Furthermore, CD4^+^ FOXP3^+^ T cells expressing IL-23R in conjunction with RORC and IL17F were identified in a Th17-like T-regulatory cell cluster in humans through single-cell RNA profiling [68]. The IL-23/IL-23R pathway has also been shown to impact the function of type 1 regulatory T cells in patients with inflammatory bowel disease, resulting in a reduction in IL-10 production [69]. Furthermore, IL-23R is also expressed on CD8^+^ T cells, where it plays a role in enhancing T cell function during immune responses. IL-23R can be induced in murine naïve CD8^+^ cells by IL-6 and TGF-β. It has been demonstrated that the IL-23/IL-23R pathway plays a role in promoting the survival, proliferation and production of inflammatory cytokines in these cells, including IFN-γ and IL-17 [70]. In certain inflammatory conditions, IL-23R signaling in CD8^+^ T cells may contribute to the exacerbation of tissue damage by driving chronic inflammation. CD8^+^ T cells expressing both IL-23R and IL-17A have been designated Tc17 cells. They have been observed in the proximity of inflamed tissues in a number of immune-mediated inflammatory diseases [71]. The number of IL-23R^+^ IL-17A^+^ CD8^+^ αβ T cells in synovial fluid from patients with psoriatic arthritis is significantly higher in comparison to healthy controls [72]. An increased prevalence of T-bet⁺ IL-23R⁺ CD8⁺ T cells was identified in the intestinal tract and peripheral blood of patients diagnosed with graft-versus-host disease (GvHD) [73]. A novel type of T cell, the gamma delta T cell (γδ T cell), is currently being investigated. These T cells possess a TCR comprising a single γ (gamma) chain and a δ (delta) chain. Both murine and human γδ T cells constitutively express IL-23R [74,75]. In lymphoid organs of naïve mice, γδ T cells constitute the predominant IL-23R-expressing subset, capable of IL-17 production in response to IL-23 stimulation. Naïve murine cells display low levels of IL-23R mRNA and protein expression in spleen γδ T cells. However, following antigen stimulation in vivo, these cells demonstrate IL-23R mRNA and protein expression. Additionally, they exhibit immunosuppressive effects on Th17 cells. These findings have been corroborated by other researchers [3,76]. It is likewise conceivable that human IL-23R^+^ γδ T cells may be involved in the pathogenesis of inflammatory disease. The capacity of γδ T cells to produce IL-17 and IL-22 in response to stimulation by IL-23 has been demonstrated. Nonetheless, in the setting of acute intestinal injury, IL-23R^+^ γδ T cells within the colonic lamina propria have been identified as the predominant source of early gut-protective IL-17A, with this production occurring in the absence of IL-23 [77,78]. Entheseal IL-23R^+^ γδ T cells expressing high levels of IL-23R transcripts, have been observed to be enriched in the joints of spondyloarthritis patients. Additionally, an increase in IL-23R^+^ γδ T cells was observed in children with primary nephrotic syndrome. A further recent discovery concerns the existence in mice and humans of a T-cell population that expresses both γδ and αβ receptors simultaneously. The cells in question display elevated levels of expression of IL-23R and demonstrate a hyperinflammatory profile when stimulated by IL-23 [3] (Figure 2).

### 4.2. Innate Lymphoid Cells

The role of IL-23R was also investigated in innate lymphoid cells (ILCs). ILCs are predominantly tissue-resident lymphoid cells that lack an antigen-specific receptor. ILCs have been identified as a heterogeneous population of lymphocytes that display cytokine and transcriptional profiles similar to those of adaptive T cells [79]. ILCs can be classified into distinct subgroups (ILC1, ILC2, and ILC3), which mirror the Th1, Th2, and Th17 subgroups of conventional T cells, respectively [80]. The IL-23R is predominantly expressed in the ILC3 subset, both in mice and in humans [81,82,83]. A Notch/RORC/IL-23R pathway has been identified as operating during the differentiation of human ILCs. The downregulation of interleukin-23 receptor (IL-23R) expression by Vitamin D on group 3 innate lymphoid cells (ILC3) and subsequent reduction in IL-23R signaling has been identified as a potential novel mechanism by which the development of intestinal inflammation may be regulated by manipulation of ILC3 [84]. It can be reasonably deduced that the aforementioned observations may assist in the formulation of protocols designed to expand the functional subpopulations of ILCs in vitro, with the ultimate objective of developing novel ILC-based therapies for diseases [85,86]. There is a broad consensus among the scientific community that ILCs are of critical importance in the maintenance of intestinal health, both within a state of equilibrium and under pathological conditions. In patients with a diagnosis of Clostridium difficile infection, there has been a noted elevation in the numbers of ILC1 and ILC3 following the administration of antibiotics [87]. Additionally, ILC3 cells in the synovial tissue of patients with spondyloarthritis demonstrate expression of RORC and IL-23R and respond to IL-23 by expressing IL-22 [3,81].

### 4.3. Myeloid Cells

The expression of IL-23R by activated granulocytes has been demonstrated in murine models of colitis or aspergillosis. However, the role of IL-23 in these cells remains to be fully confirmed [88,89]. It has been demonstrated in prior studies that there is minimal expression of IL-23R by both monocyte and dendritic cell subsets. Nevertheless, studies utilizing murine bone marrow-derived macrophages that have been stimulated via LPS and IL-10 have identified the presence of IL-23R in the absence of detectable Il12rbb. This observation indicates that IL-23R expression may potentially be induced by IFN-γ stimulation. However, further research is necessary to corroborate this hypothesis [3]. Although IL-23 is known to enhance the priming capacity of dendritic cells, there has been no further investigation into IL-23 signaling in DCs [90]. The surface expression of IL-23R was described in human monocyte-derived macrophages, which responded to IL-23 treatment with increased secretion of pro-inflammatory cytokines [91]. Furthermore, inflammatory macrophages isolated from the brains of mice with experimental autoimmune encephalomyelitis expressed IL-23R. In a house dust mite model of airway inflammation, IL-23R positive cells in the lung were mainly macrophages and CD11⁺ dendritic cells. The available data suggest that IL-23R is expressed only under specific conditions in myeloid cells [20,92]. Low levels of mRNA for IL-23R have been reported in human dendritic cells, while IL-23R levels have been found to be elevated in monocytic DCs derived from patients with HBV-related liver failure [2,93].

### 4.4. B Cells

The role of IL-23 signaling in the normal function of B cells has yet to be fully elucidated. The expression of both chains of the IL-23 receptor was demonstrated in tonsil and bone marrow plasma [94]. It has been proved that primary B cells from healthy humans do not express IL-23R. Conversely, this receptor is upregulated in acute lymphoblastic leukemia (B cells). IL-23R has previously been observed to exhibit increased expression levels in primary B-ALL cells. Moreover, IL-23 has been demonstrated to exert an inhibitory effect on tumor growth both in vitro and in vivo. This occurs through the suppression of tumor cell proliferation and the induction of apoptosis. Furthermore, studies have demonstrated that the IL-23R chain is present in chronic lymphocytic leukemia (B cells), with a positive correlation observed between this and tumor progression [95,96].

## 5. Disease Overview

**Figure 3:**
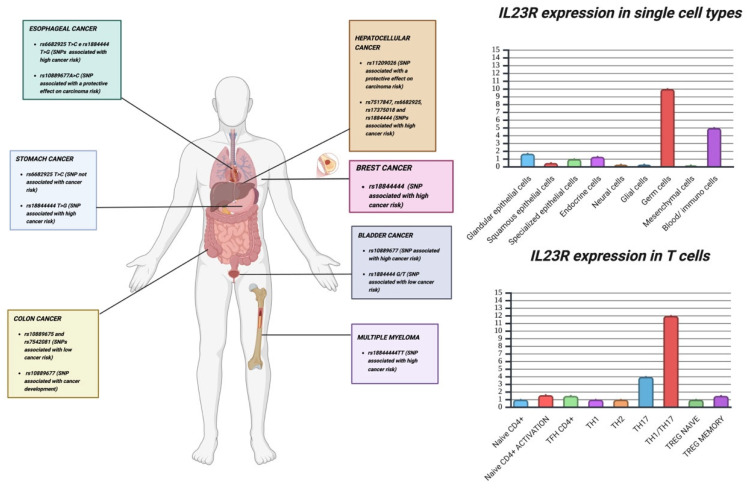
Major IL-23R-related variants in tumors and expression of IL-23R in single cells and in the T-cell lineage. The main IL-23R variants are expressed and distributed in a tumor-dependent manner. All cancers affect both genders. Breast cancer is an exception. The top panel right shows the expression of the transcript on cell types. The bottom panel right shows the relative expression of the transcript in the T lineage. Both images are freely adapted from the Human Protein Atlas. Created in Biorender. www.biorender.com/ (accessed on 20 December 2024).

Dysfunction of the IL-23/IL-23R pathway has been identified as a potential contributing factor to a number of autoimmune and chronic inflammatory conditions. In such conditions, the excessive stimulation of the IL-23R pathway gives rise to inflammatory responses that are detrimental to the affected tissue and contribute to the progression of disease. Genetic variants in IL-23R have been shown to have a robust association with the development of autoimmune disorders, with a notable prevalence observed in diseases such as ankylosing spondylitis, rheumatoid arthritis, psoriasis, and inflammatory bowel diseases (IBD) including Crohn’s disease and ulcerative colitis. Recently, there has been a significant push to gain a deeper comprehension of the pivotal function of IL-23R variants and the associated pathways in tumor growth and the surrounding tumor microenvironment. The role of IL-23R in cancer is complex, exhibiting both pro-tumorigenic and anti-tumorigenic effects depending on the circumstances. Given its dual functionality, the IL-23/IL-23R axis is being investigated as a potential target for immunotherapy in cancer, as well as a modulator that may be combined with other treatments to enhance anti-cancer immunity (Figure 3).

## 6. IL-23R Genetic Variants

Single nucleotide polymorphisms (SNPs) or single nucleotide variations (SNVs) are causal events of individual phenotypic characteristics, which result from alterations in gene expression, stability, localization, and protein function. Single nucleotide polymorphisms (SNPs) may occur within either coding or non-coding regions. Coding SNPs can be further categorized into two distinct types: synonymous and non-synonymous. A synonymous (non-coding) SNP has no effect on the protein sequence; in contrast, a non-synonymous SNP is a base exchange that occurs within an exon [97]. The majority of SNPs are located in non-coding sequences, including promoters, enhancers and introns. These can influence mRNA expression, splicing and stability. The vast number of SNPs identified for IL-23R suggests that some missense SNPs described in peer-reviewed publications may be hypomorphic, dampening IL-23R signaling or making IL-23 binding unstable [10]. The most pertinent and characterized variants are summarized in Table 1 and Figure 1A.

rs11209026 (R381Q) has been the most thoroughly investigated in terms of functional characterization. R381Q is located between the putative Jak2 binding site and the transmembrane domain within the cytoplasmic region of the IL-23R protein [3]. The R381Q mutant exhibited diminished protein stability, which led to a reduction in cellular activation by IL-23. This phenomenon was attributed to a decreased half-life of the cell-surface protein [98]. The R381Q variant has been observed to exhibit a reduction in receptor expression and a diminution in STAT3 phosphorylation in the aftermath of IL-23 stimulation. This phenomenon lends support to the hypothesis that the R381Q allele represents a variant that is inactivated in its function (loss-of-function allele) [99]. The minor allele G to A variant introduces an amino acid change at residue 381 from arginine to glutamine, which is situated in proximity to the JAK2 kinase binding site within the intracellular region of the IL-23R protein. T cells that express the minor allele demonstrate diminished IL-23-dependent STAT3 phosphorylation and IL-17 production. Furthermore, subjects who carry this variant exhibit reduced frequencies of circulating Th17 or IL-23R⁺ T cells [100,101]. The R381Q variant confers protection against ulcerative colitis and Crohn’s disease, ankylosing spondylitis and psoriasis, while increasing susceptibility to rheumatoid arthritis [102,103,104,105].

The rs1884444 (Q3H) variant indicates an amino acid exchange of a polar but uncharged amino acid for a positively charged amino acid residue in the signal peptide of IL-23R. This may impact the properties of signal peptide recognition or post-translational processing [10]. Some pathological associations have been identified, including an increased susceptibility to esophageal cancer and an elevated risk of gastric cancer [106,107]. Conversely, this variant may reduce the risk of developing ulcerative colitis and psoriasis, although it does not appear to have the same protective effect against Crohn’s disease [108].

**Figure 4:**
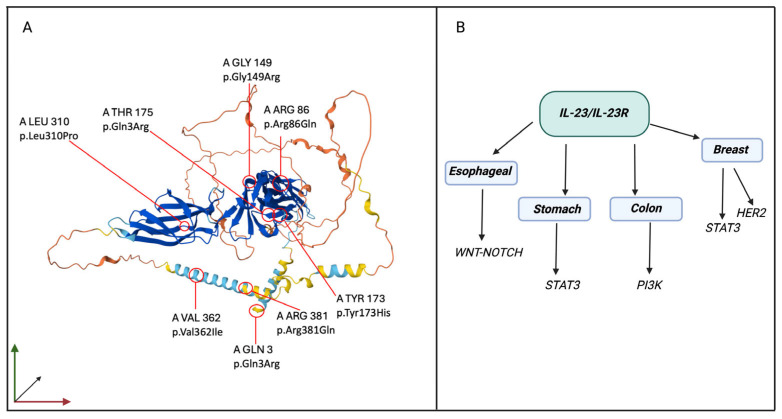
3D structure of IL-23R and pathological pathways involved in IL-23R downstream signaling in cancers. (**A**) shows the 3D structure of the IL-23R protein, with the positions of the major missense variants along the amino acid chain highlighted, according to the Alphafold database. (**B**) shows the main pathways that have been studied so far and are involved downstream of IL-23/IL-23R signaling in the above-mentioned cancers. Created in Biorender. www.biorender.com/ (accessed on 22 November 2024).

rs76575803 (R86Q) appears to exert an influence on the binding properties of IL-23R to IL-23, as evidenced by previous research [22]. The R86Q variant is situated within the extracellular domain 1. It involves the replacement of a positively charged amino acid with an uncharged polar amino acid residue. R86Q is a rare allele that has been demonstrated to confer protection against Crohn’s disease [109]. rs41313262 (V362I) is located within the transmembrane domain [109]. The V362I variation has been demonstrated to confer protection against Crohn’s disease and ulcerative colitis. This single-nucleotide polymorphism (SNP) has been shown to result in reduced protein stability, which in turn leads to reduced cell surface expression levels and diminished cellular activation by IL-23 [98,109] (Figure 4A).

**Table 1:** Major disease-related IL-23R genetic variants within various populations. The odds ratios (OR), confidence intervals (CI), clinical significance and risk value are presented for each variant. The genomic position refers to the GRCh38 genome available on ClinVar.

Variant ID	Genomic Position	Molecular Consequence	Transcript Change	Protein Change	MAF (Population)	Clinical Significance	Protective or Risk Effect	Ref.
rs41313262	chr1:67240217	missense variant	c.1084G>A	p.V362I	0.016 (European)	Benign (RCV001513561.4)	Protective against Crohn’s disease (OR = 0.548; 95% CI: 0.000–0.851) and ulcerative colitis (OR = 0.582; 95% CI: 0.000–0.854)	[109]
rs76575803	chr1:67169528	missense variant	c.257G>A	p.R86Q	0.003 (European)	Benign (RCV001521058.4)	Protective against Crohn’s disease (OR = 0.180; 95% CI: 0.000–1.125)	[109]
rs371531867	chr1:67200762	missense variant	c.517T>C	p.Y173H		Not Reported in ClinVar	No difference in functional activity compared with wild-type in transduced human T cell blasts	[110]
rs76418789	chr1:67182913	missense variant	c.445G>A	p.G149R	0.005 (European)	Benign (RCV001517127.4)	Protective against Crohn’s disease (OR = 0.230; 95% CI: 0.000–0.879) and ulcerative colitis (OR = 0.423; 95% CI: 0.000–1.146)	[109]
rs1884444	chr1:67168129	missense variant	c.9G>T	p.Q3H	0.529 (European)	Benign (RCV001520832.4)	Strong susceptibility nature in ulcerative colitis (OR = 3.13; 95% CI: 1.60–6.13) and in psoriasis (*p* = 0.005; OR = 2.68; 95% CI: 1.35–5.35)	[108]
					0.636 (Chinese)		Increased gastric cancer susceptibility (OR = 1.67; 95% CI: 1.27–2.22)	[106]
					0.636 (Chinese)		The frequency of genotype TT was higher in patients with breast tumors < 2 cm (*p* < 0.0001), whereas the frequencies of genotype GG and the G allele were higher in patients with breast tumors > 5 cm (*p* < 0.0001) and lower in Her2^+^ than in Her2^−^ patients (*p* = 0.0464)	[111]
					0.636 (Chinese)		The G allele is associated with a significant increased risk of HCC compared to the T allele (OR = 1.58; 95% CI: 0.96–2.60)	[112]
					0.636 (Chinese)		Associated with increased HCC risk in a recessive genetic model (GG vs. TT/TG: OR = 1.36, 95% CI = 1.05–1.77)	[113]
					0.645 (Japanese)		TT genotype is significantly associated with higher frequency of bone lesions (*p* = 0.04) and plasmacytoma (*p* = 0.02) than the TG and GG genotypes	[114]
rs11209026	chr1:67240275	missense variant	c.1142G>A	p.R381Q	0.061 (Global)	Protective (RCV000003254.6)	All the genetic models significantly decrease CD risk (allelic: OR = 0.42; 95% CI: 0.38–0.46; dominant: OR = 0.45; 95% CI: 0.39–0.51; recessive: OR = 0.42; 95% CI: 0.24–0.75) and UC risk (allelic: OR = 0.65; 95% CI: 0.58–0.73; dominant: OR = 0.62; 95% CI: 0.52–0.73), except for the recessive model in UC	[102]
					0.066 (European)	Protective (RCV000003255.6)	Protective against psoriasis (OR = 0.63; 95% CI: 0.50–0.79). The haplotype consisting of C at rs7530511 and A at rs11209026 has a protective effect against psoriasis (OR = 0.62; 95% CI: 0.49–0.77)	[104]
					0.066 (European)	Benign (RCV001516563.4)	Significant association with rheumatoid arthritis susceptibility in Caucasians (AA vs. GG: OR = 1.78, 95% CI = 1.02–3.10; AA vs. AG + GG: OR = 1.77, 95% CI = 1.02–3.08)	[105]
					0.013 (African)		Protective role against HCV-related HCC in Egyptian patients (OR = 0.23; 95% CI: 0.08–0.66)	[115]
rs11465797	chr1:67200769	missense variant	c.524C>A	p.T175N	0.01 (Global)	not reported in ClinVar	No association with acquired aplastic anemia	[116]
rs7530511	chr1:67219704	missense variant	c.929T>C	p.L310P	0.872 (European)	Benign (RCV001521534.4)	Protective against psoriasis (OR = 0.78; 95% CI: 0.66–0.91). The haplotype consisting of C at rs7530511 and A at rs11209026 has a protective effect against psoriasis (OR = 0.62; 95% CI: 0.49–0.77)	[104]
							The rare genotype TT showed significant association with Graves’ disease (OR = 9.4; 95% CI: 1.07–214.4)	[117]
rs10889677	chr1:67259437	intron variant			0.308 (European)	not reported in ClinVar	A allele confers increased risk of ankylosing spondylitis (OR = 1.192; 95% CI: 1.080-1.315)	[118]
					0.321 (Global)		Significantly correlated with increased risk of bladder cancer in the meta-analysis overall population using over-dominant model (OR = 1.71; 95% CI: 1.34–2.19)	[119]
rs7517847	chr1:67215986	intron variant			0.423 (European)	not reported in ClinVar	Considered to be protective factors against developing UC among Caucasian populations (OR = 0.69; 95% CI: 0.52–0.92)	[120]
					0.167 (African)		Associated with an increased risk of HCC (OR = 1.78; 95% CI: 1.21–2.62)	[121]
rs11209032	chr1:67274409	intergenic variant			0.323 (European)	not reported in ClinVar	Associated with a greater risk for UC in Caucasian populations (OR = 1.13; 95% CI: 1.00–1.26)	[120]
rs10889675	chr1:67256533	intron variant			0.115 (European)	not reported in ClinVar	Associated with decreased rectal cancer risk overall (OR = 0.68; 95% CI: 0.27–1.73) and specifically with rectal tumors bearing a TP53 mutation (OR = 0.66; 95% CI: 0.46–0.94)	[122]
rs7542081	chr1:67237570	intron variant			0.610 (European)	not reported in ClinVar	Associated with decreased rectal cancer risk overall (OR = 0.65; 95% CI: 0.45–0.92) and specifically with rectal tumors bearing a TP53 mutation (OR = 0.60; 95% CI: 0.37–0.98)	[122]
rs6682925	chr1:67165579	intron variant			0.610 (Chinese)	not reported in ClinVar	Associated with increased HCC risk in a recessive genetic model (CC vs. TT/TC: OR = 1.35, 95% CI = 1.07–1.70)	[113]
rs17375018	chr1:67189464	intron variant			0.280 (Chinese)	not reported in ClinVar	Associated with genetic susceptibility to HCC (GG vs. AA: OR = 2.324, 95%CI = 1.335–4.045; G vs. A: OR = 1.574, 95%CI = 1.211–2.045)	[123]

## 7. Cancer

The importance of IL-23R in tumor development and its influence on tumor immunity have been well documented in the scientific literature [124]. There is a discrepancy in the literature regarding whether the effects of IL-23 are pro-tumor or anti-tumor. The pro- or anticarcinogenic effects of the IL-23/IL-23R axis appear to be contingent upon several factors, including an individual’s genetic background, the specific type of tumor involved, the underlying cause (such as UV radiation, chemicals, viruses, and so forth), and the crucial equilibrium of STAT3 signaling within both the tumor mass and its surrounding microenvironment [9]. As posited by Langowski et al., the IL-23/IL-23R axis represents a significant molecular conduit between tumor-promoting pro-inflammatory processes and the failure of adaptive immune surveillance to infiltrate tumors [125]. In certain types of cancer, such as colorectal and gastric cancer, the chronic inflammation induced by the IL-23R pathway can create an environment that is conducive to tumor growth. This environment facilitates the survival, proliferation and metastasis of cancer cells. Furthermore, IL-23R signaling has the capacity to impede anti-tumor immune responses, inhibiting the activity of cytotoxic T cells and thereby facilitating tumor evasion of immune surveillance. Preclinical models of solid cancer in combination with genetic ablation of IL-23R in Treg cells have demonstrated that this particular cell type plays an essential role in mediating the tumor-promoting effects of IL-23. IL-23 represents a critical signal that drives the maintenance and stabilization of effector Treg cells by engaging with the transcription factor Foxp3 [126]. Conversely, in certain settings, IL-23R activation may enhance immune responses against tumors by promoting immune cell recruitment and activation [9]. IL-23R plays a significant role in the pathogenesis of cancer. The C allele of the rs10889677A>C polymorphism in the 3′-untranslated region of IL-23R exhibited an inverse association with the risk of multiple types of cancer, including breast, lung, and nasopharyngeal carcinoma. The rs10889677C allele was found to be associated with a significantly reduced risk of cancer compared to the rs10889677A allele in healthy controls [124]. Individuals who had not developed cancer and carried the rs10889677CC homozygous genotype demonstrated a lower proportion of regulatory T cells (Tregs) and a higher rate of T-cell proliferation than individuals who were AA homozygous for this polymorphism. The IL-23R rs10889677A>C polymorphism may exert an influence on T-cell proliferation, which in turn gives rise to modifications in the levels of Tregs in vivo and a consequent modification in cancer susceptibility [124] (Figure 3 and Figure 4B and Table 1).

### 7.1. Gastrointestinal Disease

#### 7.1.1. Esophageal Cancer

The role of the IL-23R gene in the etiology of esophageal cancer remains a topic of debate in the scientific community. Zhou et al. conducted an analysis of the effects of the IL-23/IL-23R pathway in IL-23 receptor-positive esophageal squamous cell carcinoma (ESCC IL-23R^+^). The administration of IL-23 resulted in a notable elevation in the accumulation of CD133^+^ cells and the activation of Wnt and Notch signaling pathways in ESCC CD133-IL-23R^+^ cell lines. CD133-IL-23R^+^ cells that had been subjected to IL-23 pretreatment demonstrated augmented anti-apoptotic capacity when exposed to radiation, exhibiting enhanced survival compared to the untreated control groups [127]. Activation of the IL-23/IL-23R pathway facilitates radioresistance in ESCC by triggering Wnt-Notch-mediated G0/1 phase arrest, and that attenuation of this pathway may prove an effective pretreatment for radiation therapy [127]. Two single-nucleotide polymorphisms were examined in a Chinese cohort. The IL-23R rs6682925 T>C and rs1884444 T>G variant genotypes were found to be significantly associated with an elevated risk of esophageal cancer in comparison to the corresponding wild-type homozygotes. Moreover, the elevated risk linked to the two SNPs was found to be independent of both smoking and alcohol consumption. These findings suggest a potential role for the genetic variants of IL-23R in the development of esophageal cancer [107]. Alternatively, other SNPs, such as rs10889677A>C, may exert a protective effect on the risk of esophageal squamous cell carcinoma [128]. The hypomethylation and overexpression of IL-23R were identified in esophageal adenocarcinoma, indicating that epigenetic regulation of this gene plays a pivotal role in the inflammatory cascade associated with esophageal adenocarcinoma [129].

#### 7.1.2. Stomach Cancer

With regard to gastritis, there is a paucity of reliable data and there are many conflicting opinions in the existing literature. IL-23R expression was significantly increased in stomach biopsy specimens with evidence for Helicobacter pylori infection compared with specimens negative for Helicobacter pylori infection [130]. One study demonstrated that the IL-23R^+^2199A/C polymorphism does not influence the mucosal cytokine profile in Iranian patients with H. pylori-associated gastritis [131]. However, the same polymorphism (2199A/C) has been shown to alter the mucosal expression pattern of IL-23/IL-23R in patients with gastritis in the absence of H. pylori [132]. In the context of gastric cancer, IL-23R polymorphisms were also investigated. Two of the polymorphisms (rs1884444 and rs6682925) were found to be significantly correlated with the prognosis of patients with gastric cancer [133]. However, an alternative study showed that there was no significant association between the rs6682925 T>C polymorphism and the risk of developing gastric cancer. On the other hand, the rs18844444 T>G variant has been suggested to contribute to gastric cancer susceptibility, particularly in intestinal-type gastric cancer [106]. An increased level of positive expression of IL-23R was observed in the tissues and cell lines of the gastric cancer. Compared to the adjacent normal tissue, a higher level of IL-23R expression was observed in gastric carcinoma tissues. A significant correlation was also observed between IL-23R-positive gastric cancer tissues and larger tumor size, worse T stage and poorer clinical prognosis. Binding of IL-23 to its receptor has been shown to facilitate the migration and invasion of gastric cancer cells through the induction of epithelial-to-mesenchymal transition. This occurs via the STAT3 signaling pathway [134].

#### 7.1.3. IBD and Colon Cancer

A growing body of evidence indicates that the IL-23/IL-23R axes play a pivotal role in the pathogenesis of inflammatory bowel diseases, including Crohn’s disease (CD) and ulcerative colitis (UC), as well as colon cancer [135]. Several studies have explored the expression of IL-23R and Th17-associated transcription factors in individuals afflicted with IBD, with a pronounced interest in lamina propria T cells. These investigations have demonstrated an elevation in IL-23R expression in these cells, indicating a possible role in the etiology of IBD [136,137,138]. Furthermore, Schmitt et al. [139] reported elevated levels of IL-23R mRNA and IL-23R-expressing lamina propria CD4^+^ T cells in individuals who exhibited non-response to anti-TNF therapy in Crohn’s disease (CD). Subsequent experiments indicated that the expression of the RORC transcription factor, a marker of the Th17 immune response, was elevated in lamina propria CD4⁺ T cells from individuals with CD and UC. This finding suggests the possible involvement of IL-23R-driven Th17 cells in IBD pathogenesis. However, an alternate study has indicated that, rather than RORγt, the intestinal mRNA levels of the transcription factor Batf are increased in conjunction with IL-23 mRNA levels in patients with UC, but not in those with CD [140]. The impact of genetic variants on IL-23R functionality has been the focus of scientific inquiry. A pilot study revealed an association between variants of the IL-23R gene and ileal CD. In a notable finding, an uncommon coding variant (p.Arg381Gln) was identified as a significant protective factor against the disease, while additional non-coding IL-23R variants were found to be independently associated with the risk of CD development [141]. Successive replication studies have validated these findings in independent cohorts of patients with IBD. This suggests that the risk of developing both CD and UC is influenced by IL-23R variants [142,143]. In a large-scale case–control study involving 727 patients with inflammatory bowel disease (IBD), there was a consistently observed association between the development of Crohn’s disease (CD) and the interleukin (IL)-23 receptor variant G149R. Conversely, in ulcerative colitis (UC) patients, an association was identified between G149R and Q3H [144]. An additional investigation substantiated that the IL-23R R381Q variant, situated in exon 9 and providing immunoprotective benefits against CD, was more prevalent among pediatric patients afflicted with the condition. The hypothesis put forth is that this variant may lead to a reduction in IL-23-dependent IL-17 production. Furthermore, it may impede STAT3 and Th17 activation in individuals with inflammatory bowel disease (IBD), thereby providing an explanation for the observed protective impact of this variant [145]. Despite a substantial body of evidence indicating an association between IL-23R single-nucleotide polymorphisms (SNPs) and the risk of inflammatory bowel disease (IBD) in independent patient cohorts, it is essential to acknowledge that some studies have not identified a correlation between IL-23R SNP variations and the likelihood of developing IBD. To illustrate, a study carried out in Iran on 85 patients diagnosed with ulcerative colitis (UC) demonstrated that there were no statistically significant associations between specific IL-23R polymorphisms and the onset of this condition. [146]. Therefore, it can be hypothesized that the contribution of IL-23R variants to the development of IBD displays geographical variations. For instance, two polymorphisms (rs11209026 and rs7517847) may be regarded as protective factors against the development of UC among Caucasian populations, whereas the rs11209032 polymorphism may increase the risk of UC among this ethnic group. Additionally, the rs10889677 polymorphism may be considered a protective factor against the development of UC among Asian populations [120]. Patients with IBD are at increased risk of colorectal cancer (CRC) and colitis-associated cancer (CAC). The underlying mechanism driving neoplastic progression is chronic inflammation, which can lead to dysplastic precursor lesions in different regions of the colon. This process is known as ‘field cancerization’ and is a potential consequence of chronic inflammation [135,147]. A significant number of studies have demonstrated the presence of IL-23R in human colorectal cancer tissue samples. It was discovered that all of the patients with TNM stage IV disease were positive for IL-23R, with the level of IL-23R being relatively elevated at the deepest point of invasion in a number of cases [9]. The 88413 C>A (rs10889675) and 69450 C>A (rs7542081) polymorphisms were found to be associated with an overall reduced risk of rectal cancer, particularly in cases where the tumor contained a TP53 mutation. However, none of the associations that were observed remained statistically significant after correction for multiple testing [122]. As a potential variant associated with the association between CAC, UC and CRC, the IL-23R untranslated region (UTR) variant, rs10889677, was identified. This variant plays a key role in initiating the PI3K (Phosphatidylinositol-3-Kinase) pathway, which is well documented in inflammatory cancers. Furthermore, buparlisib (an oral small molecule PI3K inhibitor) is able to inhibit non-AKT PI3K activation processes involved in CAC pathogenesis [147,148]. A significant advance has been made with regard to the creation of IL-23R-specific chimeric antigen receptor regulatory T cells (CAR Tregs) for the treatment of CD [149]. Despite the long-standing association between regulatory T cells (Tregs) and a poor prognosis in solid tumors, the role of IL-23R signaling in Tregs in colorectal cancer remains poorly characterized. The evidence would suggest that IL-23R signaling in tumor-associated regulatory T cells has opposing effects in murine models of sporadic colorectal cancer and inflammation-associated colorectal cancer. In the context of inflammation-associated colorectal cancer, there is evidence that IL-23R signaling in Treg cells has an inhibitory effect on carcinogenesis. Conversely, in sporadic colorectal cancer, IL-23R signaling in Treg cells has been observed to have a promoting effect on carcinogenesis. This illustrates that the environmental circumstances and associated conditions that give rise to colon cancer may exert disparate influences [150]. Furthermore, IL-23R^+^ Th17 have been shown to play a significant role in CRC. Studies have indicated that Batf1-dependent IL-23R^+^IL-6^+^CD4^+^ Th17 cells are crucial for regulating IL-23-driven colitis-associated tumor formation and the progression of sporadic colon tumors. Batf-dependent IL-23R^+^ T cells represent a potential avenue for further research into new treatments that could limit the progression of CRC [140].

### 7.2. Breast Cancer

Recent research into the etiology of breast cancer has focused on the role of immunity and inflammation. Gene expression levels of the *IL-23R* gene are notably higher in breast cancer tissues and positively correlated with tumor size, TNM stage and metastasis in patients. The IL-23/IL-23R pathway may therefore be a potential prognostic marker and target for the treatment of breast cancer patients [111,151]. In order to evaluate the influence of IL-23R gene polymorphisms on the susceptibility to sporadic breast cancer, a case–control study was conducted on a cohort of Chinese Han women. The *IL-23R* gene was examined for two specific single-nucleotide polymorphisms (SNPs): rs10889677, located in the 3′-untranslated region, and the non-synonymous variants (nsSNVs) rs1884444 in exon 2. The analysis included 491 breast cancer patients and 502 matched healthy controls. The findings of the analysis revealed a significant association between the rs1884444 variant in the *IL-23R* gene and the HER-2 (human epidermal growth factor receptor 2) status as well as tumor size. It is postulated that this SNP may contribute to the early development of breast cancer [111].

### 7.3. Bladder Cancer

Bladder carcinoma is a recurrent, highly aggressive neoplasm characterized by chronic, localized inflammatory processes. IL-23R has been identified as a key positive regulator of Th17 cell priming and plays an integral role in inflammatory processes contributing to neoplasia [152]. The rs10889677 “C” allele was identified as a significant risk factor for bladder cancer, with higher frequencies observed in high-grade and invasive tumors.The subsequent activation of the IL-23/17 inflammatory axis was evidenced by a clear up-regulation of IL-23R. This results in a significant elevation of IL-23R levels in the blood. Furthermore, the rs10889677 variant may be a key factor in the formation of an inflammatory environment that is more conducive to the development of malignant cells. This is due to the fact that the variant exerts its pro-tumor effects on IL-23R-bearing immune cells, including tumor-associated macrophages (TAMs), natural killer (NK) cells and CD4^+^ T-helper cells [153]. A total of 175 patients with bladder cancer who were undergoing treatment with Bacillus Calmette-Guérin immunotherapy (BCG) as a result of high-grade non-muscle invasive tumors, and 207 healthy individuals underwent genetic profiling. The IL-23R c.-309C>A allele was identified as a risk factor for bladder cancer. It may facilitate the identification of individuals who would benefit from early radical treatment [154]. Conversely, it would seem that the functional genetic variant rs1884444 G/T is significantly associated with a reduced risk of bladder urothelial carcinoma through the deregulation of the IL-23/IL-17 pathway [119].

### 7.4. Hepatocellular Carcinoma (HCC)

It has been hypothesized that variants of the *IL-23R* gene are involved in the etiology of hepatocellular carcinoma in patients with hepatitis C virus (HCV) infection. The wild-type *IL-23R* GG genotype has been identified as an important risk factor for the development of hepatocellular carcinoma. In contrast, the rare variant rs11209026 (Arg381Gln) is thought to confer protection against HCV-related hepatocellular carcinoma in Egyptian patients [115]. In the Egyptian patient cohort, the rs7517847 polymorphism has consistently been associated with an elevated risk of hepatocellular carcinoma, yet it has no discernible impact on the clinical presentation of the disease [121]. Three single-nucleotide polymorphisms in the *IL-23R* gene (rs10889677, rs1884444, and rs11465817) were investigated in a cohort of 84 patients with chronic hepatitis B, 67 patients with HBV-related liver cirrhosis, 89 patients with HBV-related hepatocellular carcinoma, and 94 healthy controls. The results indicated that subjects with the TG genotype of rs1884444 exhibited a heightened susceptibility to hepatocellular carcinoma (HCC) relative to those with the TT genotype. The rs1884444 G allele was associated with a markedly elevated risk of HCC in comparison to the T allele. The rs11465817 and rs10889677 polymorphisms of the *IL-23R* gene were not identified as being pertinent to liver disease [112]. Additional data from a Chinese cohort substantiated the hypothesis that *IL-23R* rs6682925 and rs1884444 are associated with an increased risk of hepatocellular carcinoma (HCC) in a recessive genetic model. Moreover, the variant C allele of rs6682925 in the promoter region of *IL-23R* was found to be associated with increased reporter gene activity [113]. A correlation was observed between *IL-23R* rs17375018 and genetic susceptibility to hepatocellular carcinoma (HCC). The GC haplotype was found to exhibit a significant association with risk factors linked with the disease. Additionally, the rs17375018 polymorphism was identified as being associated with portal vein tumor thrombus (PVTT) and alcohol consumption among patients with hepatocellular carcinoma (HCC). Patients with the AA genotype of the rs17375018 polymorphism exhibited a more favorable prognosis. The GG genotype of rs17375018, PVTT and TNM stage III and IV were identified as independent risk factors for HCC. The IL-23R rs17375018 polymorphism may serve as a prognostic factor in patients with HCC following interventional therapy [123]. It is noteworthy that the IL-23/IL-23R axes is not a prerequisite for the pathogenesis of non-alcoholic steatohepatitis (NASH) in preclinical models. The deletion of IL-23R does not influence the extent of liver inflammation or fibrosis observed in the NASH model [155].

### 7.5. Multiple Myeloma

The IL-23R is expressed on normal plasma cells and on multiple myeloma cells [156]. The IL-23R HH genotype (rs1884444TT) was significantly correlated with an increased incidence of bone lesions and plasmacytoma in comparison to the HQ (rs1884444TG) and QQ (rs1884444GG) genotypes. The IL-23R HH genotype was found to be significantly associated with poorer survival rates compared to the QH and HH genotypes. It is possible that IL-23R polymorphisms may affect the severity of the disease, the occurrence of bone lesions and the presence of extra-medullary disease in patients with multiple myeloma. Furthermore, IL-23R polymorphisms may contribute to an adverse prognosis in patients with MM treated with thalidomide and/or bortezomib [114].

## 8. Conclusions

In conclusion, the evidence suggests that IL-23R plays a crucial role in the interaction between the immune system and different types of cancer. This receptor governs the function of multiple immune populations, directing them, in accordance with various contexts, into pro-inflammatory or anti-inflammatory pathways. This consequently results in pro-tumor or anti-cancer outcomes. The greatest influence is observed in T cells, in particular those belonging to either the Th17 or the Treg cell cluster. The binding of IL-23 to its receptor facilitates differentiation, survival and proliferation of these cells, thereby influencing autoimmune inflammatory diseases, tumors and the tumor microenvironment.

**Figure 5:**
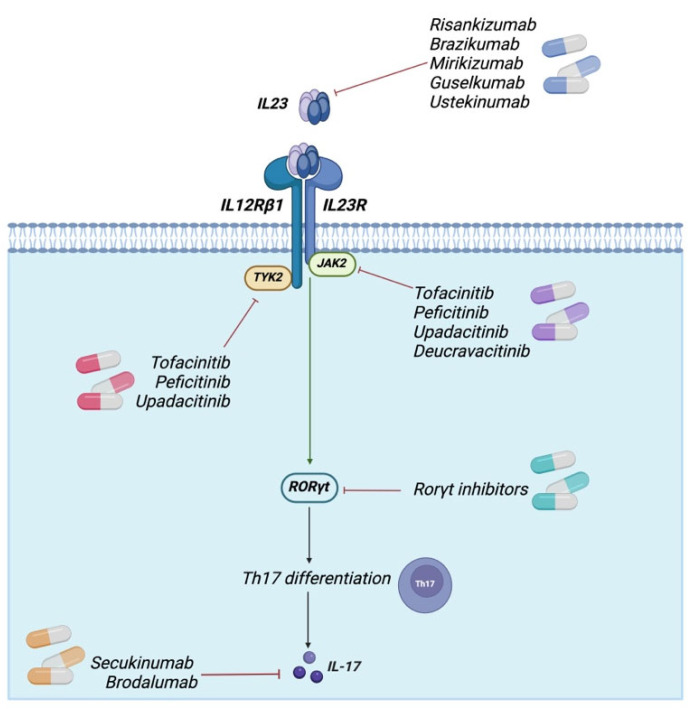
Key approved pharmacological agents for the IL-23R pathway. The figure illustrates the principal authorized pharmacological agents that interfere with IL-23R or its downstream effectors and impede the IL-23R signaling pathway. Created in BioRender. www.biorender.com/ (accessed on 20 December 2024).

Furthermore, numerous IL-23R SNPs have been identified in a multitude of cancers and inflammatory disease-related conditions, with direct or indirect consequences for the amino acid sequence. The functional characterization of the majority of IL-23R SNPs remains to be elucidated. The formation of the receptor complex remains incomplete, with several unresolved components yet to be elucidated. The collective knowledge regarding the IL-23/IL-23R configuration has facilitated the design of biological and pharmacological compounds that act as antagonists against both IL-23/IL-23R, thereby interfering with the intracellular signaling cascade that culminates in the production of deleterious mediators. The signaling of IL-23 and IL-23R can be simultaneously inhibited by ustekinumab, a monoclonal antibody that targets subunit p40. Ustekinumab received regulatory approval for the treatment of psoriasis, psoriatic arthritis, and inflammatory bowel disease (IBD). A considerable amount of research is being conducted with the objective of developing antibodies which exclusively target IL-23 via p19. Multiple anti-IL-23p19 antibodies, including risankizumab, brazikumab, mirikizumab, tildrakizumab and guselkumab, are currently being developed to target IBD [9,10]. Another example of a therapeutic agent is a protein that impairs the binding of IL-23 and IL-23R proteins. One example is an Alphabody, a protein scaffold featuring a single-chain antiparallel triple-helix coiled-coil fold, which has the ability to sequester site 3 (W156) on human IL-23. The blocking of site 3 on p19 prevents the interaction between this site and IL-23R, which in turn prevents IL-23 signaling. Moreover, the potential of small peptide antagonists to target the IL-23R with greater specificity has been assessed. It is conceivable that such alternatives may be more convenient, less cumbersome, less costly, and, most crucially, more specific than existing biologics for the treatment of inflammatory disorders [10,157,158]. A potential strategy for developing an efficacious therapy could entail combining various pharmacological agents with the objective of disrupting both extracellular cytokine-receptor assembly and intracellular signaling pathways. Furthermore, novel avenues are being explored in the domain of IL-23^+^ Tregs therapy, namely the generation of IL-23R-specific CAR Tregs for the management of Crohn’s disease [149], and the delineation of the role of Tregs in the pathogenesis of cancer. It has been demonstrated that regulatory T cells (Tregs) have the capacity to impede antitumor immunity, thereby constituting a significant impediment to the development of efficacious cancer immunotherapy. The selective targeting of regulatory Tregs that infiltrate tumors while sparing systemic Tregs represents an optimal approach to addressing this challenge. The disruption of IL-23R has been observed to result in the increased reactivity of destabilized Tregs to the cytokine IL-12, the production of γ-interferon, and the recruitment of CD8^+^ T cells that inhibit tumor growth. The combination of GITR and IL-23R antibodies in mice that had been inoculated with MC38 tumors yielded a notable increase in the efficacy of their antitumor response, as observed by the research team [159]. Accordingly, further investigation of IL-23R’s role in tumors and in cancer development seems warranted and may lead to the design of new drug profiles and innovative strategies that can alter IL-23/IL-23R signaling. Alternatively, the use of existing drugs capable of targeting the IL-23/IL-23R axes under tumor conditions may be explored (Figure 5).
